# Supplementary material for: Changes in Photosystem II Complex and Physiological Activities in Pea and Maize Plants in Response to Salt Stress
Source: Plants (Basel). 2024 Apr 3;13(7):1025. doi: 10.3390/plants13071025 (PMC11013719; doi:10.3390/plants13071025)
Supplement: Supplementary file 1 [file plants-13-01025-s001.zip › plants-2925878-supplementary.pdf]

Supplementary material

Changes in Photosystem II Complex and Physiological Activities in Pea and Maize Plants in Response to Salt Stress

Martin A. Stefanov, Georgi D. Rashkov, Preslava B. Borisova, and Emilia L. Apostolova

Table 1S. The influence of the different NaCl concentrations on the amplitudes and times of the fast (A_1, t_1) and the slow (A_2, t_2) component on the dark relaxation of chlorophyll fluorescence excited by a single saturating light in leaves of maize (*Zea mays* L. Method) and pea (*Pisum sativum* L. Ran 1). Mean values (±SE) were calculated from 8 independent measurements. Different letters indicate significant differences between the values in the same column at $p < 0.05$.

NaCl (mM)	t_1 *(s)*	t_2 *(s)*	A_1	A_2
Zea mays L.				
0	0.625±0.082[c]	12.048 ±1.306[c]	895.9 ±10.0[e]	155.5 ±1.9[c]
50	0.588 ±0.035[c]	12.195 ±1.338[c]	973.7 ±.4.7[d]	154.3 ±1.8[c]
150	0.746 ±0.022[b]	13.699 ±0.375[c]	1061.2 ±5.3[a]	136.4 ±1.7[d]
200	0.806 ±0.013[b]	14.286 ±0.204[c]	1023.7 ±5.2[bc]	126.8 ±2.0[d]
Pisum sativum L.				
0	0.422 ±.0.043[d]	14.286 ±1.224[c]	998.9 ±12.9[cd]	116.4 ±1.4[e]
50	0.380 ±.0.033[d]	12.821 ±1.479[c]	893.1 ±12.7[e]	135.9 ±1.8[d]
150	0.625 ±.0.063[c]	19.608 ±2.691[b]	769.9 ±8.5[f]	161.7 ±2.2[b]
200	1.351 ±.0.055[a]	55.556 ±3.086[a]	441.2 ±3.7[g]	199.3 ±1.70[a]

Table 2S. Variable contributions (loadings) for the principal component analysis model in Figure 1S.

Parameters	F1	F2
K_F	-0.180	-0.711
Ks	-2.955	-0.582
Af/As	0.787	-0.792
Sb	0.561	1.132
Kd	3.180	-0.526
t1	-1.316	0.734
F735/F685	-0.669	0.944
F685/F695	-0.747	-0.198

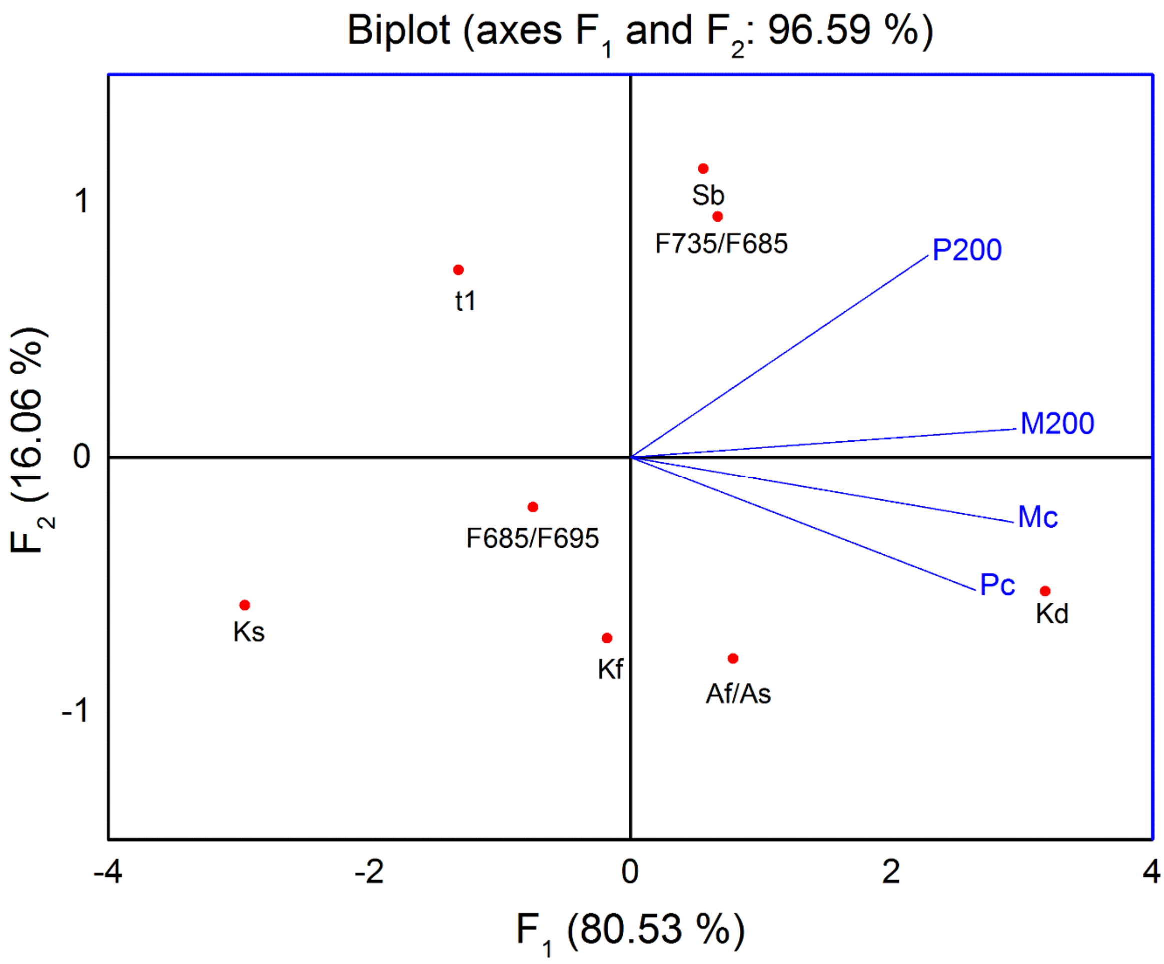

Figure 1S. Principal component analysis (PCA) shows variation within and among maize (M) and pea (P) seedlings (blue lines) in the control (Mc, Pc) and after ttreatment with 200 mM NaCl (M200, P200) in relation to the oxygen evolution (Kd, Sb, Ks, Kf), Q_A reoxidation (t1) and energy transfer within PSII (F685/F695), and between two photosystems (F735/F685) shown as red dots.
